# The pH-sensing Rim101 pathway regulates cell size in budding yeast

**DOI:** 10.1016/j.jbc.2023.102973

**Published:** 2023-02-03

**Authors:** Masaru Shimasawa, Jun-ichi Sakamaki, Tatsuya Maeda, Noboru Mizushima

**Affiliations:** 1 Department of Biochemistry and Molecular Biology, Graduate School and Faculty of Medicine, The University of Tokyo, Tokyo, Japan; 2 Department of Biology, Hamamatsu University School of Medicine, Shizuoka, Japan

**Keywords:** *Saccharomyces cerevisiae*, yeast, pH regulation, Rim101, Rim21, cell size, vacuolar ATPase, vacuole, CDS, coding sequence, TORC1, target of rapamycin complex 1, V-ATPase, vacuolar ATPase, YPD, yeast peptone dextrose

## Abstract

Although cell size regulation is crucial for cellular functions in a variety of organisms from bacteria to humans, the underlying mechanisms remain elusive. Here, we identify Rim21, a component of the pH-sensing Rim101 pathway, as a positive regulator of cell size through a flow cytometry–based genome-wide screen of *Saccharomyces cerevisiae* deletion mutants. We found that mutants defective in the Rim101 pathway were consistently smaller than wildtype cells in the log and stationary phases. We show that the expression of the active form of Rim101 increased the size of wildtype cells. Furthermore, the size of wildtype cells increased in response to external alkalization. Microscopic observation revealed that this cell size increase was associated with changes in both vacuolar and cytoplasmic volume. We also found that these volume changes were dependent on Rim21 and Rim101. In addition, a mutant lacking Vph1, a component of V-ATPase that is transcriptionally regulated by Rim101, was also smaller than wildtype cells, with no increase in size in response to alkalization. We demonstrate that the loss of Vph1 suppressed the Rim101-induced increase in cell size under physiological pH conditions. Taken together, our results suggest that the cell size of budding yeast is regulated by the Rim101–V-ATPase axis under physiological conditions as well as in response to alkaline stresses.

Although cell size varies among cell types, it is tightly controlled in each cell type (1). Many cellular processes, including the cell cycle (2), transcription (3, 4), translation (5), and metabolism (3), are size dependent. Changes in cell size are observed in senescence and pathologies such as cancers (6).

Various intracellular and extracellular factors, including cell cycle regulators and nutrients, affect cell size. Previous studies have revealed that cell size homeostasis in dividing cells is controlled at the G1/S transition, called START, by preventing cell division until a certain size is reached (7, 8). For example, *bck2*Δ, *cln3*Δ, *swi4*Δ, and *swi6*Δ cells are larger, while *whi2*Δ and *whi3*Δ cells are smaller than wildtype cells due to the dysregulation of G1 cyclins in *Saccharomyces cerevisiae* (9, 10, 11, 12, 13). Furthermore, cell size can change in response to extracellular conditions (14).

Several large-scale screens in yeast have revealed various cell size regulators, such as those involved in transcription, translation (including ribosome biogenesis), and cell cycle control (15, 16, 17, 18) (Table S1). In addition, a systematic analysis of the morphological traits of yeast deletion strains has provided cell size information (19). However, the correlations between these previous cell size screens are low (18), suggesting that additional cell size mutants were overlooked in previous screens.

Here, we conducted a genome-wide screen to identify additional cell size mutants using budding yeast. We identified the pH-sensing Rim101 pathway as a positive regulator of cell size under physiological and external alkaline conditions. Cell size increased by the activation of Rim101, and this increase was attributed to an increase in vacuolar and cytosolic volume and was mediated by the V-ATPase component Vph1. Collectively, we provide new mechanistic insight into the regulation of cell size in budding yeast.

## Results

### Identification of Rim21 as a positive regulator of cell size by genome-wide screening

**Figure 1:**
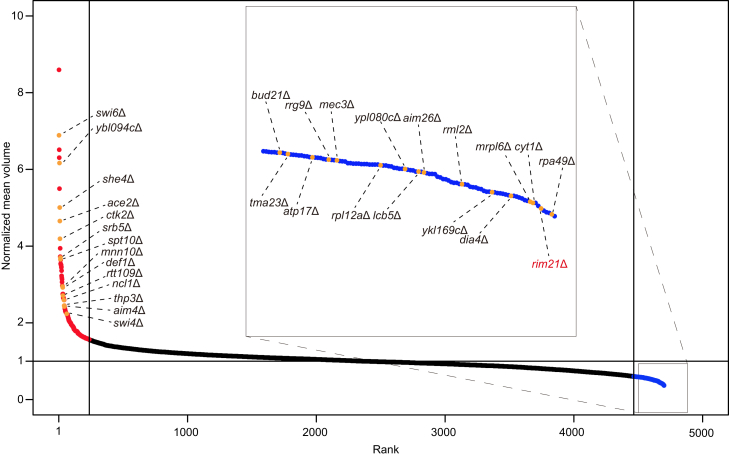
**Genome-wide screening identifies Rim21 as a positive regulator of cell size.** The cell volume of ∼4701 *S. cerevisiae* gene deletion mutants was measured. Each mutant is plotted as a dot, ranked *left* to *right* by mean volume normalized by mean volume of WT. Some known cell size mutants and *rim21*Δ are indicated. The *red* and *blue plots* show mutants ranked in the *top* 5% (*red*) and *bottom* 5% (*blue*) in size, respectively. See also Table S1.

To identify genes that regulate cell size, we performed a genome-wide screen using a nonessential gene deletion array with haploid *S. cerevisiae* strains containing ∼4782 open reading frame deletions. The cell volume was determined for 4701 mutants (excluding 81 mutants that were not grown) based on the Coulter principle. We used cells in the stationary phase to avoid the effects of bud growth. As summarized in Figure 1 and Table S1, we identified cells ranked in the top or bottom 5% (236 mutants each) for normalized mean volume as cell size mutants. In total, 102 of 236 large mutants and 51 of 236 small mutants were identified in at least one previous screen (taking the top or bottom 5% are thresholds for screens that do not annotate cell size mutants) (15, 16, 17, 18, 19, 20) (Table S1).

### The pH-sensing Rim101 pathway positively regulates cell size

**Figure 2:**
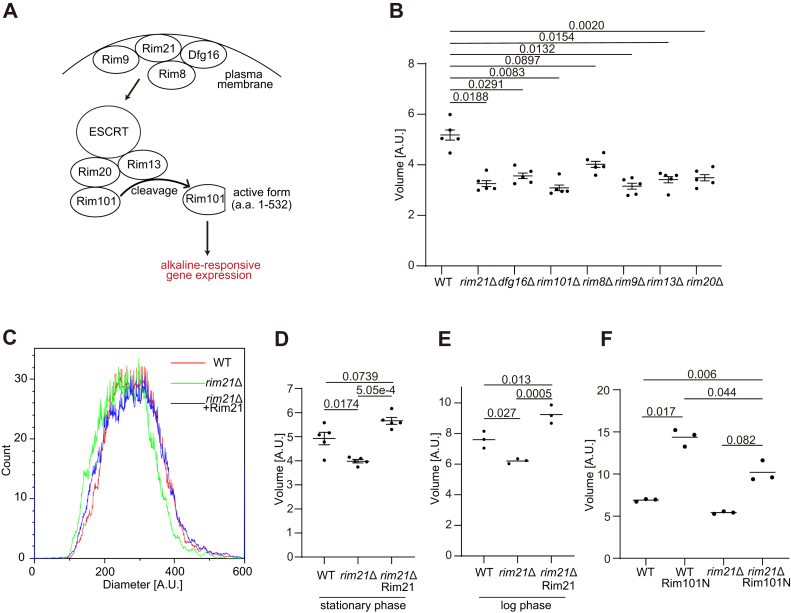
**The pH-sensing Rim101 pathway positively regulates cell size.***A*, schematic representation of the Rim101 pathway. *B*, the cell volume of wildtype (WT) and Rim101 pathway mutants in the stationary phase was measured. *C* and *D*, the cell diameter (*C*) and volume (*D*) of WT and *rim21*Δ cells reconstituted with or without Rim21 in the stationary phase was measured. The graph shows a representative result of WT and *rim21*Δ cells reconstituted with or without Rim21 (*C*). *E*, the cell volume of WT, *rim21*Δ, and *rim21*Δ reconstituted with Rim21 in the log phase was measured. *F*, the cell volume of WT and *rim21*Δ with or without the expression of the N-terminal active fragment of Rim101 (Rim101N) was measured in the stationary phase. Data represent the mean of five (*B* and *D*) or three (*E* and *F*) independent experiments. Results are plotted as individual values with mean ± standard error of the mean (SEM). Differences were statistically analyzed by one-way ANOVA and Dunnett (*B*) or Tukey (*D*–*F*) multiple comparison test. The *p* values are indicated.

Among the cell size mutants identified through our screen, we focused on *rim21*Δ because Rim21 is a well-established component of the pH-sensing Rim101 pathway (21). We noticed that other deletion mutants of the Rim101 pathway, including *rim21*Δ, *dfg16*Δ, *rim101*Δ, *rim8*Δ, *rim9*Δ, *rim13*Δ, and *rim20*Δ, showed a reduction in cell size in the screen (Table S1). The Rim101 pathway is responsible for alkaline pH–responsive gene regulation and adaptation to alkaline conditions (Fig. 2*A*) (22, 23). The complex composed of Rim9, Rim21, Dfg16, and Rim8 senses environmental alkaline pH and activates the downstream signaling pathway in an endosomal sorting complex required for transport (ESCRT) protein-dependent manner, leading to the activation of the proteolytic complex containing Rim13, a calpain-like cysteine protease, and Rim20, an adaptor for substrate recognition. Rim13 cleaves and activates the transcriptional regulator Rim101, inducing the expression of alkaline-responsive genes (Fig. 2*A*) (23). We confirmed that all Rim101 pathway mutants were significantly smaller than wildtype cells (Fig. 2*B*). In addition, we confirmed the cell size phenotype of *rim21*Δ in both the stationary and log phases (Fig. 2, *C*–*E*) and found that the expression of exogenous Rim21 restored the size of *rim21*Δ to that of wildtype cells (Fig. 2, *C*–*E*). Furthermore, the expression of the N-terminal fragment of Rim101 (1–532), the active form of Rim101 (24), increased the size of wildtype cells as well as *rim21*Δ (Fig. 2, *A* and *F*). These results suggest that the pH-sensing Rim101 pathway positively regulates cell size under physiological pH conditions.

### Cell size increases in response to external alkalization

**Figure 3:**
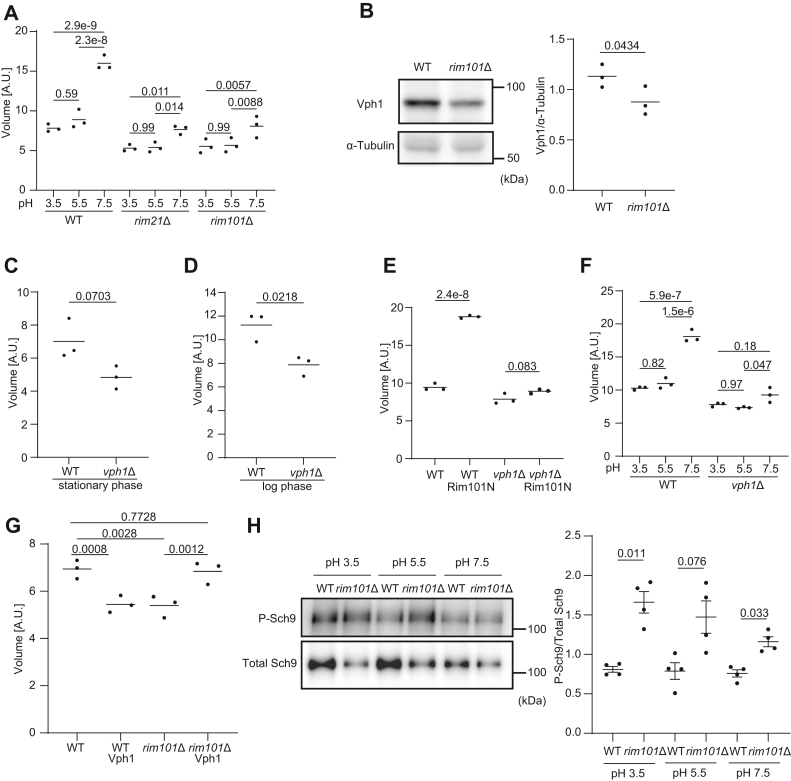
**The Rim101–V-ATPase axis positively regulates cell size.***A*, the cell volume of WT, *rim21*Δ, and *rim101*Δ cells growing in the log phase for 3 h at pH 3.5, 5.5, or 7.5 was measured. *B*, immunoblotting of Vph1 and α-tubulin and its quantification in WT and *rim101*Δ cells. *C* and *D*, the cell volume of WT and vph1Δ cells in the stationary (*C*) or log (*D*) phase was measured. *E*, the cell volume of WT and *vph1*Δ cells with or without the expression of Rim101N in the stationary phase was measured. *F*, the cell volume of WT and *vph1*Δ cells growing in the log phase for 3 h at pH 3.5, 5.5, or 7.5 was measured. *G*, the cell volume of WT and *rim101*Δ cells with and without Vph1 overexpression in the stationary phase was measured. *H*, the levels of phosphorylated (P-Sch9) and Sch9 in WT and *rim101*Δ cells at pH 3.5, 5.5, and 7.5. Lysates of cells expressing FLAG-Sch9 were subjected to immunoblotting using anti-P-Sch9 and anti-FLAG (for total Sch9) antibodies. Data represent the mean of three (*A*–*G*) or four (*H*) independent experiments. Differences were statistically analyzed by one-way ANOVA and Sidak (*A*, *F*, and *H*) or Tukey multiple (*E* and *G*) comparison test, or unpaired two-tailed Welch *t* test (*B*–*D*). The *p* values are indicated.

Because the Rim101 pathway is activated in response to external alkalization (23), we next evaluated whether external alkalization affects cell size. We found that wildtype cells were larger when they were cultured at pH 7.5 than at pH 5.5 or 3.5 (Fig. 3*A*). Note that pH 7.5 is regarded as an alkaline pH for yeast; the pH of yeast peptone dextrose (YPD), typically used to grow yeast, is approximately 6.8 (25) and becomes more acidic in the stationary phase (approximately 5.1–5.6).

We next examined whether the effect of external alkalization on cell size was dependent on the Rim101 pathway. The increase in the size of *rim21*Δ and *rim101*Δ cells in response to environmental alkalinization was largely suppressed (Fig. 3*A*). These data suggest that the Rim101 pathway positively regulates cell size under external alkaline conditions as well as under physiological pH conditions.

### The Rim101 pathway regulates cell size *via* V-ATPase

To investigate the mechanism by which the Rim101 pathway regulates cell size, we evaluated RNA sequencing data for *rim101*Δ cells (26). Vacuolar-type ATPase (V-ATPase) components, such as Vph1, were downregulated in *rim101*Δ. In addition, the deletion of *RIM101* causes the downregulation of the V-ATPase genes *VMA2* and *VMA4* (27). We thus hypothesized that the Rim101 pathway regulates cell size *via* V-ATPase expression.

We first measured the protein expression level of Vph1 in WT and *rim101*Δ cells. The amount of Vph1 was reduced in *rim101*Δ (Fig. 3*B*). These data confirmed that the Rim101 pathway positively regulates the expression of Vph1.

We found that *vph1*Δ cells were smaller than wildtype cells in both the stationary and log phases under physiological pH conditions (Fig. 3, *C* and *D*), although *vph1*Δ was not annotated as a cell size mutant in previous studies or ours (15, 16, 17, 18, 19, 20). The expression of the active form of Rim101 (1–532) failed to increase cell size in *vph1*Δ cells at physiological pH (Fig. 3*E*). Furthermore, alkalization-induced cell enlargement was suppressed in *vph1*Δ cells (Fig. 3*F*).

To investigate whether Vph1 acts downstream of the Rim101 pathway, we compared the size of wildtype and *rim101*Δ cells with and without Vph1 overexpression. The overexpression of Vph1 increased the cell size of *rim101*Δ to the level of wildtype. This supports the connection between the Rim101 pathway and Vph1 (Fig. 3*G*).

Next, we investigated how V-ATPase increases cell size. One potential mediator is target of rapamycin complex 1 (TORC1) because V-ATPase is required for TORC1 activity in response to cytosolic pH in yeast (28). We checked TORC1 activity by quantifying the level of phosphorylated Sch9 (P-Sch9) (29). However, under all pH conditions, the level of P-Sch9 relative to total Sch9 did not decrease in *rim101Δ* cells compared with WT cells (Fig. 3*H*). This suggests that TORC1 does not mediate the cell size regulation by the Rim101 pathway.

Collectively, these results suggest that the Rim101 pathway regulates cell size *via* V-ATPase under physiological conditions as well as external alkaline conditions.

### The vacuole and cytoplasm are enlarged by external alkalization *via* the Rim101-V-ATPase axis

**Figure 4:**
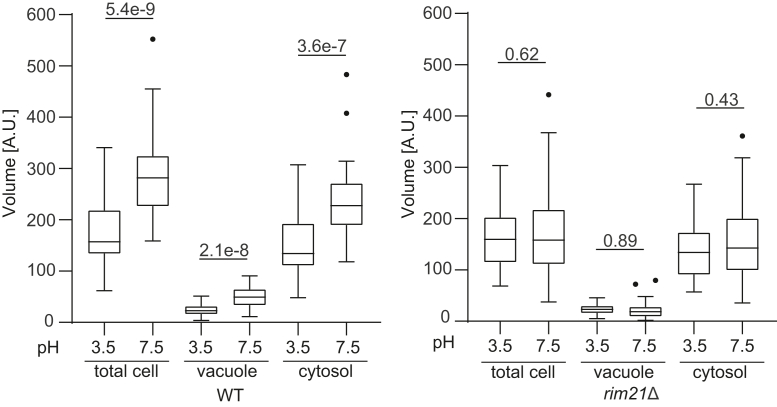
**Vacuoles are enlarged by external alkalization *via* the Rim101–V-ATPase axis.** The total cell volume and volume of the vacuole (stained with FM4-64) and cytoplasm of wildtype (WT) and *rim21*Δ cells in the log phase at pH 3.5 or 7.5 were quantified. Solid bars indicate medians, boxes indicate the interquartile range (25th to 75th percentile), whiskers indicate the largest and smallest values within 1.5 times the interquartile range, and dots represent outliers. Data were collected from 40 cells for each strain. Differences were analyzed by one-way ANOVA and Sidak multiple comparison test. The *p*-values are indicated.

Next, we asked whether cell enlargement in response to alkalization *via* the Rim101-V-ATPase axis is associated with an increase in the volume of the cytoplasm (excluding the vacuole) or the vacuole. We found that vacuolar size (as determined by FM4-64 staining) was larger in wildtype cells at pH 7.5 than at pH 3.5, and this difference was abolished in *rim21*Δ cells (Fig. 4). In addition, an increase in the volume of the cytoplasm was also observed in wildtype cells at pH 7.5 but not in *rim21*Δ cells. These results suggest that both the vacuole and cytoplasm are enlarged by external alkalization *via* the Rim101-V-ATPase axis (Fig. 4).

## Discussion

**Figure 5:**
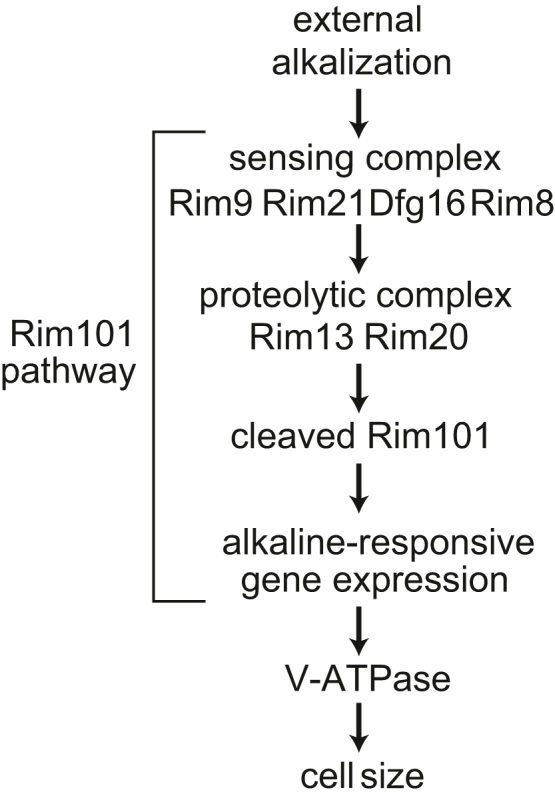
**Schematic representation of the Rim101–V-ATPase axis.** Integrated model of cell size regulation by the Rim101 pathway. Rim101 is cleaved and translocated to the nucleus in response to external alkalization, which triggers alkaline-responsive gene expression, including the increased expression of V-ATPase components, thereby increasing cell size by altering both the vacuolar and cytoplasmic volumes.

We identified Rim21, a component of the pH-sensing Rim101 pathway as a positive regulator of cell size by a genome-wide screen. Subsequently, we confirmed that other mutants defective in the Rim101 pathway were also smaller than wildtype cells. Consistent with the role of the Rim101 pathway in pH sensing, we found that cell size increases in response to external alkalinization in a manner dependent on the Rim101 pathway and V-ATPase. Based on these findings, we propose that the size of budding yeast cells is regulated by the Rim101-V-ATPase axis under both physiological and external alkaline conditions (Fig. 5).

Although the Rim101 pathway is activated in response to external alkalization, the Rim101 pathway mutants were also smaller around pH 5.5. This may be attributed to the basal activity of the Rim101 pathway; the processed active form of Rim101 can be detected in cells in the log phase cultured in YPD medium (30). The increase in the active form under alkaline conditions (23) is consistent with the increased effect of Rim101 deficiency on cell size under those conditions. We also found that the increase in cell size in response to alkaline conditions is not completely abolished in *rim21*Δ and *rim101*Δ cells (Fig. 3*A*), suggesting that other pH-sensing mechanisms contribute to this increase.

The mechanism by which V-ATPase increases cell size is unclear. A simple explanation is that V-ATPase-mediated proton translocation increases intravacuolar osmolarity and causes vacuolar swelling. This effect would be enhanced under alkaline conditions to maintain acidity in vacuoles. In fact, V-ATPase activity in cells is higher at pH 7 than at pH 5 (28).

Cell size regulation in response to the external pH by the Rim101 pathway is not likely to be conserved in mammals. In mammals, calpain-7 and ALIX are possible orthologs of yeast Rim13 and Rim20, respectively (23). These mammalian orthologs are not thought to function in ambient pH sensing and adaptation (23). However, it is possible that the cell size under alkaline conditions in mammals is regulated *via* V-ATPase, which regulates mTORC1 under some conditions in mammals (31).

In *Cryptococcus neoformans*, *rim101*Δ cells are smaller than wildtype cells in host mice (32). However, the size of *rim101*Δ cells does not differ from that of wildtype cells *in vitro*. The phenotype of *rim101*Δ in *C. neoformans* can likely be explained by a decrease in titan cells (characterized by enlarged size) and the small size of minimally encapsulated fungal cells. In *Escherichia coli*, environmental pH impacts the length at which cells divide and therefore cell size increases as the pH rises (33). Thus, the mechanism underlying cell size regulation in *S. cerevisiae* appears to differ from those of bacteria. Furthermore, the cell sizes of *Staphylococcus aureus* (33), *Streptococcus pneumoniae* (34), and *Caulobacter crescentus* (35) increase during growth in alkaline media. It is conceivable that cell size regulation in response to environmental pH has a common physiological role across taxa, despite differences in the underlying mechanisms.

## Experimental procedures

### Antibodies and reagents

For immunoblotting, anti-FLAG (Sigma-Aldrich, Cat#: F1804), anti-Vph1 antibody (abcam, Cat#: ab113683), anti-α-tubulin (Sigma-Aldrich, Cat#: T9026), anti-phospho-T737-Sch9 (36), horseradish peroxidase (HRP)-conjugated anti-mouse IgG (Jackson ImmunoResearch Laboratories, Cat#: 111-035-003), and anti-rabbit IgG (Jackson ImmunoResearch Laboratories, Cat#: 111-035-144) antibodies were used.

### Yeast strains

A deletion library of *S. cerevisiae* in the BY4741 background (MATa *his*Δ*1 leu2*Δ*0 met15*Δ*0 ura3*Δ*0*) (37) was purchased from the Yeast Knockout Collection (Transomic Technologies, Cat# TKY3502).

### Plasmids

The *RIM21* gene (including the region 1 kb upstream of the coding sequence [CDS], CDS, and 0.3 kb downstream of the CDS) was amplified by PCR using BY4741 genomic DNA as a template and inserted into the pRS316 vector (38). The N-terminal region of RIM101 (encoding Met1–Gln532) was amplified by PCR using BY4741 genomic DNA and inserted into the pRS316 vector containing the glyceraldehyde-3-phosphate dehydrogenase promoter, 3×FLAG tag, and phosphoglycerate kinase terminator. The *VPH1* gene was amplified by PCR using pRS316-scVph1 (provided by Dr Takayuki Sekito) and inserted into the pRS316 vector containing the glyceraldehyde-3-phosphate dehydrogenase promoter.

### Yeast media

YPD medium (1% yeast extract [Becton, Dickinson and Company, Cat# 212750] with 2% bactopeptone [Becton, Dickinson and Company, Cat#: 211677] and 2% glucose [Nacalai Tesque, Cat#: 16805-35]) was used for yeast culture. After transformation with pRS316 plasmids, yeast cells were cultured in synthetic defined medium (0.17% yeast nitrogen base without amino acids and ammonium sulfate [Becton, Dickinson and Company, Cat#: 233520], 0.5% ammonium sulfate [Nacalai Tesque, Cat#: 02619-15], and 2% glucose supplemented with 0.5% casamino acids [Becton, Dickinson and Company, Cat#: 223050], 0.02 mg/ml adenine [Sigma-Aldrich, Cat#: A3159], and 0.02 mg/ml l-tryptophan [Fuji Film, Cat#: 204-03382]). For experiments under different pH conditions, YPD was buffered by using 50 mM morpholinepropanesulfonic acid (Mops [Dojindo, Cat#: 341-08241]) and 50 mM morpholineethanesulfonic acid (Mes, [Nacalai Tesque, Cat#: 21623-26]) and adjusted to pH 3.5 with HCl or to pH 5.5 or 7.5 with NaOH.

### Yeast transformation

Yeast transformation was performed by a standard method as described (39).

### Measurements of cell size

Volumes and diameters of approximately 10,000 cells were determined using an EC800 cell analyzer (Sony Japan). Data were processed using Kaluza (Beckman Coulter). The normalized mean volume, used as a proxy for cell size, was calculated by dividing the mean volume of each mutant by that of the wildtype.

### Immunoblotting

A volume of 3 ml of yeast cell cultures in the log phase grown in YPD medium or YPD medium buffered at pH 3.5, 5.5, and 7.5 was collected by centrifugation at 12,000*g* for 1 min at 4 °C. The pellets were mixed with 140 μl alkaline solution (0.3 M NaOH, 100 mM dithiothreitol [Nacalai Tesque, Cat#: 14112-52]) containing protease inhibitor cocktail [Nacalai Tesque, Cat#: 03969-34]). The samples were then vortexed and incubated on ice for 5 min followed by the addition of 1 ml of ice-cold 15% trichloroacetic acid solution (Wako, Cat#: 200-08085). The samples were incubated on ice for 10 min and centrifuged at 15,000*g* for 5 min at 4 °C. The pellets were washed in 600 μl of ice-cold acetone with sonication for 2 min and centrifuged at 15,000*g* for 5 min at 4 °C.

The pellets were then mixed with sample buffer solution (Nacalai Tesque, Cat#: 09499-14) and heated at 55 °C for 10 min. The samples were run on SDS-PAGE gel using Tris-glycine-SDS buffer, transferred to Immobilon-P polyvinylidene difluoride membranes (IPVH00010; EMD Millipore) and subjected to immunoblotting using the antibodies described above. Immobilon Western Chemiluminescent HRP Substrate (P90715; EMD Millipore Corporation) or Super-Signal West Pico Chemiluminescent Substrate (1856135; Thermo Fisher Scientific) was used to visualize the signals. The signals were detected using a Fusion Solo 7S system (M&S Instruments Inc). Images were adjusted using Adobe Photoshop 2022 (Adobe). Quantitation of signal intensity was performed using Fiji (40).

### Fluorescence imaging and quantification of the vacuolar size

Cells were incubated with 30 nM FM4-64FX (Thermo Fisher Scientific, Cat#: F34653) at 30 °C for 20 min, washed, and resuspended in 5 ml of YPD. The cells were further incubated at 30 °C for 90 min. Images were acquired on the FV3000 (Olympus) equipped with a UAPON 100XOTIRF lens (Olympus, Cat# N2709500, NA 1.49) and processed using Fiji software (40). The outline of each cell was determined based on phase-contrast observations. The major axes of the cells and their vacuoles were measured manually using Fiji software. The cube of the major axes was used as a proxy for the total cell and vacuolar volume. The volume of cytoplasm was estimated by subtracting the vacuolar volume from the total cell volume.

### Statistical analysis

The Welch *t* test was used for two-group comparisons. Multiple comparisons were performed by one-way analysis of variance followed by Dunnett test, Sidak multiple comparison test, or Tukey multiple comparison test using GraphPad Prism 8 (GraphPad Software). Normal distributions were assumed but not formally tested.

## Data availability

All data supporting the analyses described in the article are available from the corresponding author upon reasonable request.

## Supporting information

This article contains supporting information (15, 16, 17, 18, 19, 20).

## Conflict of interest

The authors declare that they have no conflicts of interest with the contents of this article.

## References

[1] Ginzberg M.B., Kafri R., Kirschner M. (2015). Cell biology. On being the right (cell) size. Science.

[2] Amodeo A.A., Skotheim J.M. (2016). Cell-size control. Cold Spring Harb. Perspect. Biol..

[3] Miettinen T.P., Pessa H.K., Caldez M.J., Fuhrer T., Diril M.K., Sauer U. (2014). Identification of transcriptional and metabolic programs related to mammalian cell size. Curr. Biol..

[4] Padovan-Merhar O., Nair G.P., Biaesch A.G., Mayer A., Scarfone S., Foley S.W. (2015). Single mammalian cells compensate for differences in cellular volume and DNA copy number through independent global transcriptional mechanisms. Mol. Cell.

[5] Marguerat S., Schmidt A., Codlin S., Chen W., Aebersold R., Bahler J. (2012). Quantitative analysis of fission yeast transcriptomes and proteomes in proliferating and quiescent cells. Cell.

[6] Neurohr G.E., Terry R.L., Lengefeld J., Bonney M., Brittingham G.P., Moretto F. (2019). Excessive cell growth causes cytoplasm dilution and contributes to senescence. Cell.

[7] Fantes P.A., Grant W.D., Pritchard R.H., Sudbery P.E., Wheals A.E. (1975). The regulation of cell size and the control of mitosis. J. Theor. Biol..

[8] Hartwell L.H. (1974). Saccharomyces cerevisiae cell cycle. Bacteriol. Rev..

[9] Tyers M., Tokiwa G., Futcher B. (1993). Comparison of the Saccharomyces cerevisiae G1 cyclins: Cln3 may be an upstream activator of Cln1, Cln2 and other cyclins. EMBO J..

[10] Nash R., Tokiwa G., Anand S., Erickson K., Futcher A.B. (1988). The WHI1+ gene of Saccharomyces cerevisiae tethers cell division to cell size and is a cyclin homolog. EMBO J..

[11] Dirick L., Bohm T., Nasmyth K. (1995). Roles and regulation of Cln-Cdc28 kinases at the start of the cell cycle of Saccharomyces cerevisiae. EMBO J..

[12] Di Como C.J., Chang H., Arndt K.T. (1995). Activation of CLN1 and CLN2 G1 cyclin gene expression by BCK2. Mol. Cell Biol..

[13] Sudbery P.E., Goodey A.R., Carter B.L. (1980). Genes which control cell proliferation in the yeast Saccharomyces cerevisiae. Nature.

[14] Turner J.J., Ewald J.C., Skotheim J.M. (2012). Cell size control in yeast. Curr. Biol..

[15] Jorgensen P., Nishikawa J.L., Breitkreutz B.J., Tyers M. (2002). Systematic identification of pathways that couple cell growth and division in yeast. Science.

[16] Zhang J., Schneider C., Ottmers L., Rodriguez R., Day A., Markwardt J. (2002). Genomic scale mutant hunt identifies cell size homeostasis genes in S. cerevisiae. Curr. Biol..

[17] Dungrawala H., Hua H., Wright J., Abraham L., Kasemsri T., McDowell A. (2012). Identification of new cell size control genes in S. cerevisiae. Cell Div..

[18] Soifer I., Barkai N. (2014). Systematic identification of cell size regulators in budding yeast. Mol. Syst. Biol..

[19] Ohya Y., Sese J., Yukawa M., Sano F., Nakatani Y., Saito T.L. (2005). High-dimensional and large-scale phenotyping of yeast mutants. Proc. Natl. Acad. Sci. U. S. A..

[20] Hoose S.A., Rawlings J.A., Kelly M.M., Leitch M.C., Ababneh Q.O., Robles J.P. (2012). A systematic analysis of cell cycle regulators in yeast reveals that most factors act independently of cell size to control initiation of division. PLoS Genet..

[21] Obara K., Yamamoto H., Kihara A. (2012). Membrane protein Rim21 plays a central role in sensing ambient pH in Saccharomyces cerevisiae. J. Biol. Chem..

[22] Lamb T.M., Xu W., Diamond A., Mitchell A.P. (2001). Alkaline response genes of Saccharomyces cerevisiae and their relationship to the RIM101 pathway. J. Biol. Chem..

[23] Maeda T. (2012). The signaling mechanism of ambient pH sensing and adaptation in yeast and fungi. FEBS J..

[24] Rockenfeller P., Smolnig M., Diessl J., Bashir M., Schmiedhofer V., Knittelfelder O. (2018). Diacylglycerol triggers Rim101 pathway-dependent necrosis in yeast: a model for lipotoxicity. Cell Death Differ..

[25] Wasko B.M., Carr D.T., Tung H., Doan H., Schurman N., Neault J.R. (2013). Buffering the pH of the culture medium does not extend yeast replicative lifespan. F1000Res.

[26] Read T., Richmond P.A., Dowell R.D. (2016). A trans-acting variant within the transcription factor RIM101 interacts with genetic background to determine its regulatory capacity. PLoS Genet..

[27] Perez-Sampietro M., Herrero E. (2014). The PacC-family protein Rim101 prevents selenite toxicity in Saccharomyces cerevisiae by controlling vacuolar acidification. Fungal Genet. Biol..

[28] Dechant R., Saad S., Ibanez A.J., Peter M. (2014). Cytosolic pH regulates cell growth through distinct GTPases, Arf1 and Gtr1, to promote Ras/PKA and TORC1 activity. Mol. Cell.

[29] Urban J., Soulard A., Huber A., Lippman S., Mukhopadhyay D., Deloche O. (2007). Sch9 is a major target of TORC1 in Saccharomyces cerevisiae. Mol. Cell.

[30] Diakov T.T., Kane P.M. (2010). Regulation of vacuolar proton-translocating ATPase activity and assembly by extracellular pH. J. Biol. Chem..

[31] Zoncu R., Bar-Peled L., Efeyan A., Wang S., Sancak Y., Sabatini D.M. (2011). mTORC1 senses lysosomal amino acids through an inside-out mechanism that requires the vacuolar H(+)-ATPase. Science.

[32] O'Meara T.R., Holmer S.M., Selvig K., Dietrich F., Alspaugh J.A. (2013). Cryptococcus neoformans Rim101 is associated with cell wall remodeling and evasion of the host immune responses. mBio.

[33] Mueller E.A., Westfall C.S., Levin P.A. (2020). pH-dependent activation of cytokinesis modulates Escherichia coli cell size. PLoS Genet..

[34] Perez A.J., Cesbron Y., Shaw S.L., Bazan Villicana J., Tsui H.T., Boersma M.J. (2019). Movement dynamics of divisome proteins and PBP2x:FtsW in cells of Streptococcus pneumoniae. Proc. Natl. Acad. Sci. U. S. A..

[35] Heinrich K., Leslie D.J., Morlock M., Bertilsson S., Jonas K. (2019). Molecular basis and ecological relevance of Caulobacter cell filamentation in freshwater habitats. mBio.

[36] Takahara T., Maeda T. (2012). Transient sequestration of TORC1 into stress granules during heat stress. Mol. Cell.

[37] Brachmann C.B., Davies A., Cost G.J., Caputo E., Li J., Hieter P. (1998). Designer deletion strains derived from Saccharomyces cerevisiae S288C: a useful set of strains and plasmids for PCR-mediated gene disruption and other applications. Yeast.

[38] Sikorski R.S., Hieter P. (1989). A system of shuttle vectors and yeast host strains designed for efficient manipulation of DNA in Saccharomyces cerevisiae. Genetics.

[39] Gietz R.D., Schiestl R.H. (2007). Quick and easy yeast transformation using the LiAc/SS carrier DNA/PEG method. Nat. Protoc..

[40] Schindelin J., Arganda-Carreras I., Frise E., Kaynig V., Longair M., Pietzsch T. (2012). Fiji: an open-source platform for biological-image analysis. Nat. Met..

